# Effects of rikkunshito on quality of life in patients with gastroesophageal reflux disease refractory to proton pump inhibitor therapy

**DOI:** 10.3164/jcbn.16-77

**Published:** 2017-02-08

**Authors:** Takashi Kawai, Yoji Hirayama, Aiko Oguchi, Fumi Ishii, Masanao Matushita, Naoya Kitayama, Shinji Morishita, Noboru Hiratsuka, Ken Ohata, Hiroyuki Konishi, Maiko Kishino, Shinichi Nakamura

**Affiliations:** 1 Gastroenterological Endoscopy, Tokyo Medical University, 6-7-1 Nishishinjuku, Shinjuku-ku, Tokyo 160-0023, Japan; 2 Department of General Medicine and Primary Care, Tokyo Medical University Hospital, 6-7-1 Nishishinjuku, Shinjuku-ku, Tokyo 160-0023, Japan; 3 Shinjuku NS Building Clinic, 2-4-1 Nishishinjuku, Shinjuku-ku, Tokyo 163-0820, Japan; 4 Shinjuku Mitsui Building Clinic, 2-1-1 Nishishinjuku, Shinjuku-ku, Tokyo 183-0410, Japan; 5 Department of Internal Medicine, International Catholic Hospital, 2-5-1 Nakaochiai, Shinjuku-ku, Tokyo 161-8521, Japan; 6 Department of Gastroenterology, Tokyo Shinjuku Medical Center, 5-1 Tsukudo-cho, Shinjuku-ku, Tokyo 162-0821, Japan; 7 Hiratsuka Gastroenterological Hospital Shinjuku Center Clinic, 1-25-1 Nishishinjuku, Shinjuku-ku, Tokyo 163-0690, Japan; 8 Department of Gastroenterology, NTT Medical Center Tokyo, 5-9-22 Higashi-gotanda, Shinagawa-ku, Tokyo 141-8625, Japan; 9 Department of Gastroenterology, Tokyo Women’s Medical University Hospital, 8-1 Kawada-cho, Shinjuku-ku, Tokyo 162-8666, Japan

**Keywords:** quality of life, gastroesophageal reflux disease, rikkunshito, proton pump inhibitor

## Abstract

We investigated the effects of rikkunshito, in combination with a proton pump inhibitor, on symptoms and quality of life in patients with proton pump inhibitor-refractory gastroesophageal reflux disease. The subjects were 47 patients with gastroesophageal reflux disease with residual symptoms such as heartburn following 8 weeks of proton pump inhibitor therapy. We administered these subjects rikkunshito in combination with a proton pump inhibitor for 6–8 weeks. We scored their symptoms of heartburn, fullness, abdominal discomfort, and abdominal pain, and surveyed their quality of life using the Reflux Esophagitis Symptom Questionnaire, comprising questions concerning daily activities, meals (changes in amount and favorite foods), and sleep (getting to sleep and early morning waking). Improvement was seen in all symptoms, and quality of life scores for meals and sleep also improved. These results indicate that combination therapy with rikkunshito and a proton pump inhibitor improves quality of life related to eating and sleep in patients with patients with proton pump inhibitor-refractory gastroesophageal reflux disease.

## Introduction

In recent years, we have seen changes in the pattern and distribution of gastrointestinal diseases in Japan, in association with a reduced prevalence of *Helicobacter pylori* (*H. pylori*) infection and the Westernisation of the Japanese diet. Manabe *et al.*^(1)^ investigated changes in the pattern of gastrointestinal diseases in the 1980s, 1990s and 2000s, reporting that endoscopic findings revealed increased prevalence of gastroesophageal reflux disease (GERD) and negative endoscopic findings (NERD), and a decreased prevalence of peptic ulcers. GERD is becoming a problem because it does not only cause heartburn and other symptoms, but also adversely affects quality of life (QOL). Recently it is reported that persistent reflux symptom related to mental health and sleep disorder.^(2)^ Whether there is endoscopic evidence of esophageal mucosal damage or not, significant impairment of QOL is reported in patients with heartburn symptoms at least once weekly,^(3)^ whereas amelioration of symptoms with proton pump inhibitor (PPI) therapy has been shown to improve QOL.^(4)^

Consensus has been obtained in Japan and overseas for PPI therapy as the pharmacotherapy of first choice for GERD, both as initial and maintenance therapy. The Japanese Society of Gastroenterology “Therapeutic Guidelines for Gastroesophageal Reflux Disease (GERD)” also give PPI therapy a Grade A recommendation.^(5)^ Recently multicenter prospective study verified PPIs had effectiveness for severe reflux esophagitis.^(6)^ However, some 40–50% of patients with non-erosive reflux disease (NERD), and 6–15% of those with erosive esophagitis (EE), are reported to be refractory to PPI therapy.^(7)^ In a study conducted with subjects with EE, examining the relationship between increasing the PPI dosage to increase the percentage of time with intragastric pH≥4, and improvement in symptoms, GERD symptoms improved as the percentage of time with intragastric pH≥4 as far as 50–75%, but no further improvement was seen as the percentage of time exceeded 75%.^(8)^ In other words, these results suggest the existence of a limit to improvement in GERD symptoms through the suppression of gastric acid secretion alone. It is a matter of course that QOL is impaired in patients with PPI-refractory GERD,^(9)^ so new treatments are required for this patient group.

Miwa *et al.*^(10)^ reported that rikkunshito, a Kampo (traditional Japanese) medicine, improved the barrier function of the esophageal mucosa in rats with experimental reflux esophagitis, and may stabilize symptoms. Recently, Tominaga *et al.*^(11)^ reported that rikkunshito in combination with a PPI is useful in improving symptoms in PPI-refractory GERD patients. In this study, we investigated the effect of PPI + rikkunshito combination therapy on heartburn and other symptoms, as well as QOL, in PPI-refractory GERD patients.

## Methods

This study was a multicentre collaborative trial, conducted between September 2010 and June 2011 at 9 medical centers in Shinjuku Prefecture in Tokyo. The subjects were 47 patients with residual GERD symptoms such as heartburn despite at least 8 weeks of PPI therapy, either rabeprazole (RPZ) 20 mg/day, lansoprazole (LPZ) 30 mg/day, or omeprazole (OPZ) 20 mg/day. Their average age was 65.4 ± 9.2 years, with a male:female ratio of 1.35:1. Their PPI was continued, with the addition of rikkunshito (7.5 g t.i.d) for 6–8 weeks, and subjects were surveyed about changes in their symptoms and QOL. Subjects 20 years or younger, and those with a history of gastrointestinal surgery, including the oesophagus and stomach, were excluded from this study.

The study protocol conformed to the 1975 Helsinki Declaration concerning human experiments, as revised in 1983. Informed consent was obtained from all subjects.

Subjects underwent esophagogastroduodenoscopy (EGD) prior to commencement of rikkunshito, to check for the presence and degree of reflux esophagitis (RE), and the presence of other upper gastrointestinal disease.

Symptoms of heartburn, fullness, abdominal discomfort, and abdominal pain were scored using a 5 step visual analogue scale (VAS). QOL was surveyed using the Reflux Esophagitis Symptom Questionnaire (RESQ) produced by Hongo *et al.*,^(12)^ concerning daily activities, meals (changes in amount and favorite foods), and sleep (getting to sleep and early morning waking), and similarly scored using a 5 step scale.

Statistical analyses were performed using the SPSS^®^16.0J for Windows Base System analytical software package (IBM SPSS, Chicago, IL). Changes in the RE symptom VAS and RESQ scores were analyzed using Wilcoxon’s rank test. A *p* value <0.05 was considered statistically significant.

## Results

The PPI used was RPZ for 20 subjects, LPZ for 18, and OPZ for 9. The subjects’ mean BMI was 22.6 ± 2.7. The endoscopic findings were minimal in most cases, with LA classification grade A in 3 subjects, and grade M or N in all the rest. Mean symptom VAS scores at the time of registration were 1.85 for heartburn, 1.93 for fullness, 2.46 for abdominal discomfort, and 1.30 for abdominal pain, with abdominal discomfort the most severe symptom. The QOL scores were 1.30 for daily activities, 1.74 for meals: changes in amount, 2.21 for meals: favorite foods, 2.81 for sleep: getting to sleep, and 2.64 for sleep: early morning waking, with the greatest decline in QOL seen for daily activities.

Symptom scores following the addition of rikkunshito were 1.26 for heartburn, 1.26 for fullness, 1.48 for abdominal discomfort, and 0.65 for abdominal pain, with improvement seen in all symptoms (Table 1). The QOL scores were 2.19 for daily activities, 2.40 for meals: changes in amount, 2.57 for meals: favorite foods, 2.83 for sleep: getting to sleep, and 2.81 for sleep: early morning waking, with improvement seen in all parameters except getting to sleep (Table 2).

## Discussion

GERD is divided into EE and NERD. Although NERD was initially considered a mild version of EE, recent studies have demonstrated that NERD and EE are separate conditions. Epidemiologically, EE is characterized as being more common in males, with a greater body mass index (BMI), esophageal hiatal hernia, with minimal atrophic changes. Contrastingly, NERD is more common in females, with a lesser BMI, no hiatal hernia, and strong atrophic changes.^(13)^

Although endoscopic evaluation is not essential for the diagnosis of GERD, EGD should always be performed in cases of PPI-refractory GERD, as possible causes include malignancies such as esophageal and gastric cancers, and organic diseases such as eosinophilic esophagitis. It is also important to consider the possibility of functional esophageal disorders such as achalasia.

Cases of abnormal esophageal reflux despite PPI therapy have been reported due to a lack of PPI efficacy.^(14)^ The cause is thought to be due to differences the phenotype of CYP2C19, the metabolizing enzyme for PPIs. In rapid extensive metabolizers, little suppression of gastric acid secretion by PPIs is actually seen.^(15)^ Furthermore, acid secretion may be suppressed during the day, but reduction in the intragastric pH at night (nocturnal acid breakthrough: NAB) may be seen.^(16)^

One factor other than gastric acid that can play a role in GERD is bile. Heartburn symptoms are also reported when bile acid is introduced into the esophagus.^(17)^ The mechanism of heartburn caused by bile acid is thought to involve enlargement of the intercellular space diameter in the esophageal mucosa, increasing esophageal sensitivity.^(18,19)^

Rikkunshito improves the symptoms of GERD by enhancing esophageal clearance and reducing esophageal acid exposure time.^(20)^ Furthermore, Takeda *et al.*^(21)^ and Yakabi *et al.*^(22)^ reported that rikkunshito enhances ghrelin secretion, thereby improving esophageal and gastric motility. Kusunoki *et al.*^(23)^ reported improved gastric adaptive relaxation, and Tatsuta *et al.*^(24)^ reported improved gastric emptying, indicating that rikkunshito ameliorates GERD symptoms through effects on not only the esophagus, but also the stomach.

In addition to symptomatic improvement, in this study we found that RESQ scores improved for almost all QOL parameters. The MOS Short-Form 36 item Health Survey (SF-36), the SF-8, and the Euro QOL are used to assess QOL in GERD patients, but these questionnaires do not include questions related to sleep or meals, both characteristically impaired by GERD, and there is no standard QOL survey instrument for GERD patients. RESQ and the SF-8 correlate moderately well, and the RESQ scores are low when typical GERD symptoms are severe, confirming the discriminatory validity of the RESQ survey.^(25)^ Tominaga *et al.*^(26)^ reported that RPZ + rikkunshito combination therapy improves QOL in PPI-refractory GERD patients. Using the RESQ survey in this study, we demonstrated that the addition of rikkunshito also improved QOL related to meals and sleep.

The effects of rikkunshito in the treatment of PPI-refractory GERD involve multiple factors and are not confined to effects on gastric acid secretion. It is clear that RPZ + rikkunshito combination therapy improves QOL in this patient group, and is indicated in all with GERD refractory to PPI monotherapy.

## Figures and Tables

**Table 1:** Mean symptom VAS scores with reference to registration

	Pretreatment	Post-treatment	
Heartburn	1.85 ± 1.52	1.13 ± 1.33	*p*<0.001
Fullness	1.93 ± 1.53	1.26 ± 1.16	*p*<0.001
Abdominal discomfort	2.46 ± 1.38	1.48 ± 1.09	*p*<0.001
Abdominal pain	1.30 ± 1.63	0.65 ± 1.12	*p* = 0.001

Mean symptom VAS scores of registration were 1.85 for heartburn, 1.93 for fullness, 2.46 for abdominal discomfort, and 1.30 for abdominal pain before the addition of rikkunsito. Symptom scores following the addition of rikkunshito were 1.26 for heartburn, 1.26 for fullness, 1.48 for abdominal discomfort, and 0.65 for abdominal pain, with improvement seen in all symptoms with abdominal discomfort the most severe symptom.

**Table 2:** Score of QOL was surveyed using the Reflux Esophagitis Symptom Questionnaire (RESQ)

	Pretreatment	Post-treatment	
Difficulty in daily activities	1.30 ± 0.81	2.19 ± 0.90	*p*<0.001
Dissatisfaction with limitation in eating amount	1.74 ± 0.85	2.40 ± 0.90	*p*<0.001
Dissatisfaction with having to avoid favorite foods	2.21 ± 1.00	2.57 ± 1.02	*p*<0.01
Dissatisfaction with difficulty falling asleep	2.81 ± 0.80	2.83 ± 0.82	*p* = 0.563
Dissatisfaction with interrupted sleep	2.64 ± 0.76	2.81 ± 0.71	*p* = 0.035

The QOL scores were 1.30 for daily activities, 1.74 for meals: changes in amount, 2.21 for meals: favorite foods, 2.81 for sleep: getting to sleep, and 2.64 for sleep before the addition of rikkunsito. The QOL scores were 2.19 for daily activities, 2.40 for meals: changes in amount, 2.57 for meals: favorite foods, 2.83 for sleep: getting to sleep, and 2.81 for sleep: early morning waking, with improvement seen in all parameters except getting to sleep.
